# Evaluating the Impact of a Novel Mobile Care Team on the Prevalence of Ambulatory Care Sensitive Conditions Presenting to Emergency Medical Services in Nova Scotia

**DOI:** 10.7759/cureus.37280

**Published:** 2023-04-08

**Authors:** Ryan Brown, Judah Goldstein, Jan L Jensen, Andrew H Travers, Alix Carter

**Affiliations:** 1 Interprofessional Practice & Learning, Nova Scotia Health Authority, Sydney, CAN; 2 Emergency Medicine, Dalhousie University, Halifax, CAN

**Keywords:** prevalence, primary care, community paramedicine, ambulatory care sensitive conditions, emergency medical services

## Abstract

Introduction: Hospitalization due to ambulatory care sensitive conditions (ACSC) is a proxy measure for access to primary care. Emergency Medical Services (EMS) are increasingly called when primary care cannot be accessed. A novel paramedic-nurse EMS Mobile Care Team (MCT) was implemented in an under-serviced community. The MCT responds in a non-transport unit to referrals from EMS, emergency and primary care, and to low-acuity 911 calls in a defined geographic region. Our objective was to compare the prevalence of ACSC in ground ambulance (GA) responses before and after the introduction of the MCT.

Methods: A cross-sectional analysis of GA and MCT patients with ACSC (determined by chief complaint, clinical impression, treatment protocol, and medical history) from one year pre-MCT implementation to one year post-MCT implementation was conducted for the period of October 1, 2012, to September 30, 2014. Demographics were described. ACSC prevalence was compared using the chi-squared test.

Results: There were 975 calls pre-MCT and 1208 GA/95 MCT calls post-MCT. ACSC in GA patients pre- and post-MCT was similar: n=122, 12.5% vs. n=185, 15.3%; p=0.06. ACSC in patients seen by EMS (GA plus MCT) increased in the post-MCT period: 122 (12.5%) vs. 204 (15.7%) p=0.04. Pre-MCT implementation vs post-implementation, GA ACSC calls differed significantly by sex with higher female utilization (n=50 vs. n=105; p=0.007), but not age (65.38, ± 15.12 vs. 62.51 ± 20.48; p=0.16)*.* Post-MCT, the prevalence of specific ACSC increased for GA: hypertension (p<0.001) and congestive heart failure (p=0.04). MCT patients with ACSC were less likely to have a primary care provider compared to GA (90.2% and 87.6% vs. 63.2%; p=0.003, p=0.004).

Conclusion: The prevalence of ACSC did not decrease for GA with the introduction of the MCT, but ACSC in the overall patient population served by EMS increased. It is possible more patients with ACSC call, or are referred to EMS, for the new MCT service. Given that MCT patients were less likely to have a primary care provider, this may represent an increase in access to care or a shift away from other emergency/episodic care. These associations must be further studied to inform the ideal utility of adding such services to EMS and healthcare systems.

## Introduction

Ambulatory care sensitive conditions (ACSC) were first defined by Billings and colleagues in 1993 as “chronic medical conditions that when treated effectively in community settings should not, in most cases, advance to hospitalizations” (p.165) [1]. Based upon this definition, ACSC hospitalizations have been used as a proxy measure for inadequate primary care and primary care access [2-5]. 

While a number of conditions are included as ACSC internationally [6,7], as this study takes place in the Canadian setting, the acceptable list based on the World Health Organization’s International Disease Classification (ICD) codes as adopted by the Canadian Institute for Health Information (CIHI) are used [2]. This list includes seven conditions: epileptic seizures, chronic obstructive pulmonary disease (COPD), diabetes, hypertension (HTN), angina, and congestive heart failure (CHF) (and other pulmonary edema). All diseases of cardiac origin exclude cardiac procedures [2].

Paramedics are trained to treat critically ill patients and most commonly work within Emergency Medical Services (EMS) systems designed to respond to emergencies. Despite this, patients with primary care complaints access EMS when they have challenges obtaining care elsewhere [8]. Community paramedicine has evolved in response to this phenomenon [9]. Community paramedics have an expanded scope or role in managing patients with non-emergent medical issues, minor injuries, and exacerbations of chronic conditions, often in the home without transport to an emergency department [10]. In some services, they work in partnership with other healthcare providers. 

In May of 2013, a cohort of experienced paramedics and emergency certified Registered Nurses (RNs) were trained for the Mobile Care Team (MCT) in the town of New Waterford, Nova Scotia, to respond to low acuity emergency and primary care complaints. This model was based on a similar team in the United Kingdom (UK) as described by Widiatmoko et al. (2008) [11], and consists of a specially outfitted sport utility vehicle (2010 Ford Expedition; Ford Motor Company, Dearborn, Michigan, United States) in which the team presents to patients. The team can be activated via a number of methods and may treat and discharge, treat and discharge with follow-up, or transfer to a regional facility [12].

The MCT was implemented on October 1st, 2013. The UK model was studied for cost-effectiveness, in which it showed potential, but safety and specific conditions treated were not considered [11]. While a paucity of research exists on the safety of such models, work that has been done in this area typically uses rates of EMS callback at various intervals and subsequent hospitalization as an outcome measure [13,14].

Our objective was to determine the prevalence of ACSC in ground ambulance (GA) responses before and after the introduction of a novel care delivery system (MCT) in the community of New Waterford, Nova Scotia, in order to assess the impact of the MCT on ACSC in the community.

This article was previously presented as a meeting abstract at the 2016 Canadian Association of Emergency Physicians (CAEP) Annual Scientific Meeting on June 5, 2016 [15].

## Materials and methods

The methodology for this study has been previously published [12]. In brief, this was a longitudinal, cross-sectional, comparative study utilizing secondary data from an electronic health records database. Two consecutive years, one before implementation (October 1, 2012, to September 30, 2013) and another after implementation (October 1, 2013, to September 30, 2014) were compared. Data were reported in accordance with the Strengthening the Reporting of Observational Studies in Epidemiology (STROBE) guideline [16]. 

Sampling

Convenience sampling of patients presenting with ACSC complaints to EMS in New Waterford, Nova Scotia, Canada, was accessed via the electronic patient care record (ePCR). The ePCR contains paramedic-documented patient history, assessment, diagnosis, and treatment. All patients presenting to ground ambulance with ACSC conditions during the year prior to MCT implementation (October 1, 2012-September 30, 2013), patients presenting to the MCT in the year following implementation (October 1, 2013-September 30, 2014), and patients presenting to ground ambulance in the year following implementation of MCT (October 1, 2013-September 30, 2014) were included. Patients presenting with any complaint other than ACSC were excluded. This study was approved by the Capital District Health Authority Research Ethics Board, Halifax, Nova Scotia, Canada (approval number: 2015-253).

Outcomes

The primary outcome measure was the prevalence of ACSC in EMS responses. ACSC is a group of conditions often used as a proxy for the quality of, and access to, primary care. They may be sub-acute or acute but do not require admission to hospital [17]. The pathologies included as ACSC vary from list to list. The list and definitions utilized in this study are that of Sanmartin and colleagues (2011) [2], as developed for the CIHI.

Data collection

Both clinical and demographic data were collected from the ePCR database. Clinical data collected included chief complaint, past medical history, clinical impression, and prehospital protocol followed. Demographic data collected were age, gender, and whether the case had a primary care provider. A review of 10% of the charts was completed by the lead investigator (RB) ensuring that data points were captured accurately.

ACSC complaints were determined by the charted chief complaint and clinical impression. If there was still ambiguity, past medical history (PMHx) and care protocol selected by the paramedic and/or nurse was investigated. ACSC complaints were further subdivided into pathology as per CIHI criteria.

Pilot testing

A full chart review of 10% of the charts was conducted to ensure the variables in the data matrix (as described in a previously published methods paper) was, in fact, capturing the desired data [12]. Numerical values 1-326 were assigned to all ACSC master incident numbers (MIN). A web-based number randomizer, Research Randomizer v4.0 [18] was utilized to randomly select 10% (n=33) MINs for review. Charts were accessed via the secure ePCR database. All charts were found to be ACSC calls as per the paramedic and/or nurse’s narrative, signs and symptoms, and treatment, thus validating the data matrix as no adjustments were necessary.

Data analysis

Frequency tables were constructed for the variable of age and contingency tables for the variables of primary care provider and sex for each of the included time frames and response types. Ages were reported as a mean with standard deviation (SD). Mean ages were compared by using Student’s t-test. Primary care provider and sex were reported as proportions and were compared pre- and post-MCT implementation via Pearson’s chi-squared test.

The prevalence of ACSC based on this data was calculated and expressed as percentages and proportions. These proportions were also compared pre- and post-MCT implementation via Pearson’s chi-squared test for the included time periods as well as cumulatively (pre-MCT ground ambulance responses vs. post-MCT ground ambulance responses and MCT responses). This was then repeated for each of the seven CIHI ACSC conditions as a subgroup analysis. All analysis was conducted using IBM SPSS Statistics for Windows, Version 21.0 (Released 2012; IBM Corp., Armonk, New York, United States) and imported from the cleaned Microsoft Excel spreadsheets (Microsoft Corporation, Redmond, Washington, United States).

## Results

Demographics

**Figure 1:**
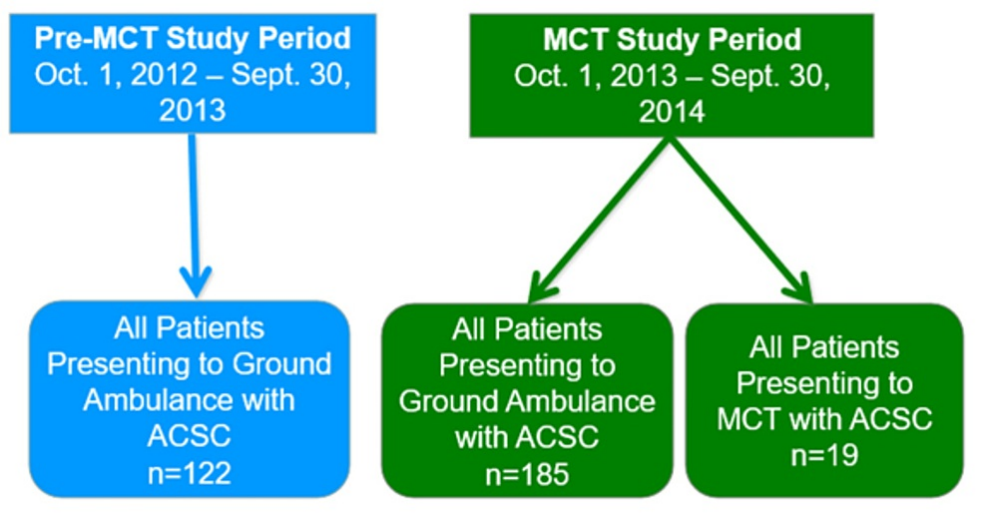
Included participants MCT: mobile care team; ACSC: ambulatory care sensitive conditions

**Figure 2:**
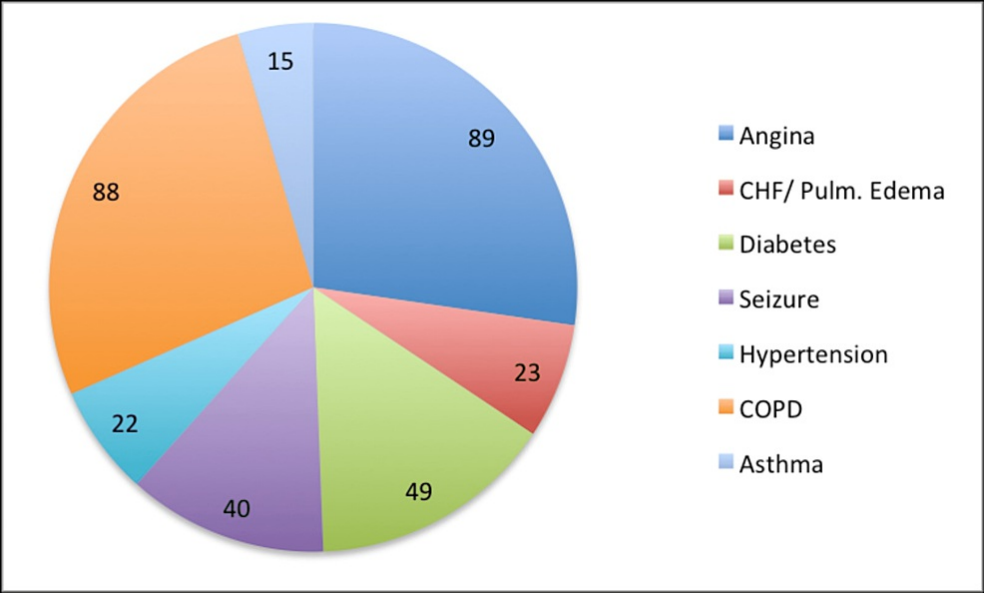
Frequency of individual ACSC pathologies in New Waterford, Nova Scotia, as presenting to EMS from October 1, 2012, to September 30, 2014 (n=326) CHF: congestive heart failure; Pulm. Edema:  pulmonary edema; COPD:  chronic obstructive pulmonary disease; ACSC: ambulatory care sensitive conditions; EMS: Emergency Medical Service

Ground ambulance patients during the pre-MCT time frame, were 59.0% (72/122) male compared to 43.2% (80/185) in the post-MCT period. The majority of ground ambulance patients in the pre-MCT and post-MCT periods had a primary care provider listed. The mean age was 65.38 ± 15.12 years pre-MCT and 62.51 ± 20.48 years post-MCT. The MCT patients had a mean age of 69.84 ± 15.52 years, were largely attached to a primary care provider (12/19; 63.2%), and were mostly female (12/19; 63.2%). The total number of ACSC presenting to EMS during the study period was 326 (see Figure 1 for included participants and Figure 2 for frequency of individual ACSC).

Pearson’s chi-squared analysis did not demonstrate a significant difference in sex with regards to the pre-MCT and MCT time interval (Chi^2^=3.28, p=0.07) or between the post-MCT and MCT interval (Chi^2^=0.29, p=0.59). A significant difference was, however, demonstrated between the pre-MCT and post-MCT intervals (Chi^2^=7.32, p=0.007).

A significant difference was not demonstrated with regard to the primary care provider during the pre-MCT and post-MCT interval (Chi^2^=0.49, p=0.48). There was, however, a significant difference demonstrated pre-MCT implementation vs post-implementation (Chi^2^=8.94, p=0.003) and post-MCT GA vs MCT (Chi^2^=8.19, p=0.004) with a lower percentage of patients (63.2%) attended to by the MCT having a primary care provider listed (Table 1). ACSC prevalence for the pre-MCT time period was calculated to be 12.5%, with post-MCT ACSC cumulative prevalence of 15.7% (15.3% ground ambulance, 20% MCT) and a total of 14.3% over the two-year period (Table 2).

**Table 1:** Summary of ACSC patient demographics MCT: mobile care team; SD: standard deviation; ACSC: ambulatory care sensitive conditions

Response Type	Male		Female		Primary Care Provider		No Primary Care Provider		Mean Age (years)
	n	%	N	%	n	%	n	%	
Pre-MCT Ground Ambulance	72	59.0	50	41.0	110	90.2	12	9.8	65.38 (SD 15.12)
Post-MCT Ground Ambulance	80	43.2	105	56.8	162	87.6	23	12.4	62.51 (SD 20.48)
MCT	7	36.8	12	63.2	12	63.2	7	36.8	69.84 (SD 15.52)

**Table 2:** ACSC prevalence in New Waterford, Nova Scotia as presenting to EMS from October 1, 2012 to September 30, 2014 EMS; emergency medical service; ACSC: ambulatory care sensitive conditions

Period in Time	Response Type	ACSC Complaints	Other Complaints	Prevalence
October 1, 2012 – September 30, 2013	Ground Ambulance	n=122	n=975	12.5%
October 1, 2013 – September 30, 2014	Ground Ambulance	n=185	n=1208	15.3%
October 1, 2013 – September 30, 2014	Mobile Care Team	n=19	n=95	20.0%
October 1, 2013 – September 30, 2014	Ground Ambulance & Mobile Care Team	n=204	n=1303	15.7%
October 1, 2012 – September 30, 2014	Ground Ambulance & Mobile Care Team	n=326	n=2278	14.3%

Overall ACSC prevalence

Pre-MCT and post-MCT comparison of ground ambulance ACSC prevalence (122/975 and 185/1208, respectively) did not yield a significant difference (Chi^2^=3.50, p=0.06), thus accepting the null hypothesis that the MCT has not impacted the prevalence of ACSC ground ambulance responses. However, there was a significant difference when the pre-MCT period was compared with the cumulative ACSC prevalence of both ground ambulance and the MCT post implementation (204/1303) (Chi^2^=4.09, p=0.04), showing a higher prevalence of ACSC during the latter period. This was also true when comparing pre-MCT implementation ground ambulance prevalence of ACSC and MCT prevalence (19/95) of ACSC (Chi^2^=4.24, p=0.04). When comparing the post-MCT interval to the MCT response only, there was no significant difference (Chi^2^=1.46, p=0.23). Subgroup prevalence analysis was also conducted and can be found in Table 3.

**Table 3:** Comparison of ACSC prevalence (%) between pre-MCT implementation and post-MCT implementation ground ambulance responses ACSC: ambulatory care sensitive conditions; MCT: mobile care team; CHF: congestive heart failure; COPD: chronic obstructive pulmonary disease

ACSC	Pre-MCT Ground Ambulance Prevalence		Post-MCT Ground Ambulance Prevalence		Significance
	n	%	n	%	
Total ACSC	122	12.5	185	15.3	p=0.06
CHF/Pulmonary Edema	<5	<1.0	15	1.2	p=0.04
Angina	46	4.7	41	3.4	p=0.12
Seizure	12	1.2	28	2.3	p=0.06
Diabetes	21	2.2	27	2.2	p=0.80
Hypertension	0	0.0	15	1.2	p<0.001
COPD	35	3.6	48	4.0	p=0.64
Asthma	<5	<1.0	11	0.9	p=0.16

## Discussion

The implementation of the MCT did not reduce ground ambulance EMS responses during the year following its launch. There was no statistically significant difference in prevalence with the year prior to launch (12.5% vs. 15.3%). There was, interestingly enough, a statistically significant overall increase in ACSC prevalence when the MCT prevalence was pooled with the ground ambulance data (12.5% vs. 15.7%). This is of interest as the alternative hypothesis developed from the outset of the study was that the implementation of the MCT would decrease the prevalence of ACSC seen by ground ambulance. This was not accomplished by the MCT; however, the pooled data show an increase, which is counter-intuitive as the target population of the MCT is low-acuity patients with primary care complaints, many of which tend to be ACSC related. This can be explained statistically by the fact that the MCT did see high rates of ACSC (20%); however, it would be expected this would ease the burden of these complaints on the ground ambulance counterparts.

While this was not anticipated, it seems this phenomenon is not uncommon. Two community paramedic models in Nova Scotia, Collaborative Emergency Centres (CEC) and the Extended Care Paramedic (ECP) program, had experienced similar results. When first implemented, the CECs saw a spike in patient presentations, which gradually declined around the six-month mark [19]. During the study conducted by Jensen et al. (2013) on the ECP program, data was also collected showing an increase in call volume for end-of-life care and other complaints once the long-term care facilities were aware of the ECP unit’s capabilities [13]. The implementation of the MCT in the community of New Waterford was a highly publicized and contentious issue as the unit was instituted to deal with primary healthcare issues at night due to the closure of the ED during these hours. To help alleviate tensions, the government held a number of community meetings and a public relations campaign. The increased usage of the MCT for ACSC conditions may be due to the fact that the community was made aware of what the unit was capable of; thus, it was called for preferentially by patients who otherwise would not have sought medical attention. Anecdotally, ground ambulance paramedics report patients would often ask for the MCT when they arrived on the scene if the MCT was providing care elsewhere at the time of the call. It is, however, possible there were simply more calls for ACSC as a whole during this period as opposed to the pre-implementation period. To account for these potential confounders, matching based on ACSC past medical history and propensity matching could be employed.

While no difference was found between ground ambulance prevalence of ACSC between the pre-MCT implementation and post-MCT implementation periods, the sub-group analysis did demonstrate a statistically significant increase in the presentation of two individual ACSC to ground ambulance in the post-implementation period; CHF/pulmonary edema (<1.0% vs. 1.2%) and HTN (0% vs. 1.2%). The MCT also saw a high prevalence of these pathologies at 4.2% and 7.4%, respectively. 

Again, it could be postulated that this is due to public awareness of the MCT, specifically for HTN as there were no ground ambulance calls for HTN in the pre-MCT period but there were 15 out of 1208 in the post-MCT implementation period. An increase in call volume due to public awareness is also displayed in the EMS literature for such pathologies as myocardial infarction and stroke [20,21]. It is important to note that in the case of HTN specifically, Walker et al. (2014) found hypertensive patients tended to utilize primary care providers more often than other pathologies and with increased frequency leading up to hospitalizations [22]. Due to this fact, it is plausible that increased HTN prevalence in the EMS setting was a convenient alternative to seeing a primary care provider for this complaint. This, however, also discredits HTN hospitalizations as a proxy for poor primary care access [22].

Concerning the demographic factors analyzed, there was no association found when looking at sex although some of the broader literature cites males as having higher rates of ACSC [23]. However, the definition of ACSC used in this particular study was not that of Sanmartin et al. (2011) [2]. In Canada, there have been no high-quality data generated on sex differences in ACSC; however, this has been called for by CIHI [24]. Similarly, a paucity of information exists in Canada on age differences for ACSC hospitalizations; however, some studies do point to those of more advanced age suffering from more ACSC [23].

When analyzing the variable of primary care provider, there was only a statistically significant association when comparing the pre-MCT implementation and post-MCT implementation ground ambulance responses to the MCT itself. Guttmann et al. (2010) demonstrated that within the Canadian context (a universal system), access to care consists of more than simply having a provider [25]. There can also be issues with physical access to care due to geography or socioeconomic constraints. Taking this into account the fact that MCT did show a significant decrease in those patients with providers, the MCT actually showing up to the home may be bridging access even for those with providers, thus amplifying its effect on access to the patient population overall. This is an important finding as access to care is limited when no primary care provider is available to manage ACSC.

Limitations

Two limitations of the study were the hours of operation of the MCT as well as the diagnostic accuracy of paramedics/nurses for the various ACSC complaints. Due to the necessity of a physician or nurse practitioner being available for next-day referral if needed, the MCT only ran four nights per week (Monday-Thursday, 1900-0700 hours) as there were staffing issues with these practitioners. While this limitation does not affect the raw prevalence calculations, if the MCT were running seven nights a week there may be more impact demonstrated on ground ambulance prevalence (whether that be an increase or a decrease). Furthermore, the model studied by Widiatmoko et al. (2008) also ran four days a week for a 15-week pilot and yielded meaningful data on conveyance rates to the hospital, albeit not in the context of ACSC [11].

Concerning the diagnosis of patients, the CIHI uses ICD codes, which are typically abstracted via chart review upon discharge in ACSC studies [2,23]. ICD codes are a standardized tool and represent the gold standard in clinical diagnoses, which are most often, but not in all cases, made by a physician [26,27]. While paramedics have been shown to diagnose some conditions such as sepsis with similar specificity and accuracy to physicians [28], it is reasonable to estimate that not all pre-hospital working diagnoses were correct as paramedics and nurses have less education than physicians and do not have the diagnostic tools available in the community that would be available to their hospital-based colleagues. It is worth noting, however, that each patient encounter was a mandatory consult with an online physician and the clinical impression in the chart would more than likely reflect the physician’s impression.

## Conclusions

We found no difference in ground ambulance ACSC prevalence following the implementation of the MCT; however, a broader look at the data showed that ACSC prevalence did increase overall in the community. Furthermore, there were significant increases in the prevalence of ground ambulance presentations of two specific ACSC, HTN and CHF/pulmonary edema. While this small sample in the community of New Waterford does not yield results that can impact a solution for the public health problem of access to care, it has uncovered positive associations which require study on a larger scale.
